# Effect of Vertebral Body Tether Tensioning on Vertebral Body Growth Modulation

**DOI:** 10.1002/jsp2.70098

**Published:** 2025-07-24

**Authors:** Taylor J. Jackson, Craig Louer, Ron El‐Hawary, Jennifer Hurry, Hui Nian, Christine Farnsworth, Carrie E. Bartley, Tracey P. Bryan, Michael P. Kelly, Peter O. Newton, Stefan Parent, Pediatric Spine Study Group, Vidyadhar V. Upasani

**Affiliations:** ^1^ Division of Orthopedics & Scoliosis Rady Children's Hospital San Diego California USA; ^2^ Department of Orthopedics Vanderbilt University Medical Center Nashville Tennessee USA; ^3^ IWK Health Center Halifax Nova Scotia Canada; ^4^ Department of Orthopaedics University of California San Diego California USA; ^5^ St. Justine University of Montreal Montreal Canada; ^6^ Pediatric Spine Foundation Valley Forge Pennsylvania USA

**Keywords:** 3D spine reconstruction, biplanar slot scanning radiograph, growth modulation, scoliosis, vertebral body tether

## Abstract

**Background:**

The relationship between tether tension and spinal growth modulation following vertebral body tethering (VBT) has not been studied in growing children.

**Aims:**

This study aims to explore the relationship between vertebral body growth modulation under varying tether tension.

**Materials and Methods:**

A retrospective, multicenter pediatric registry was queried for idiopathic scoliosis patients treated with right‐sided VBT, with recorded intraoperative tension (using an ordinal scale of 0–3), and 3D reconstructions from biplanar radiographs at the first erect (FE) and 2‐year post‐operative visits. Custom MATLAB code was used to calculate vertebral height (mm) on the untethered and tethered sides from T5‐T12. Generalized linear mixed models were used to analyze the effect of tension on vertebral body growth.

**Results:**

Fifty‐two subjects (47 female) were included with a mean age of 12.5 ± 1 years. Patients were skeletally immature (triradiate cartilage open in 23 patients) with Proximal Femoral Maturity Index scores of 2 (nine patients), 3 (21 patients), 4 (20 patients), and 5 (two patients). A total of 330 vertebral bodies were analyzed. Mean height change of the vertebral bodies from FE to 2 years was 1.6 + 1.9 mm (untethered) and 1.2 + 1.8 mm (tethered). On the tethered side, greater tension resulted in less height change, with the greatest differential growth observed between maximal tension and no tension (0.8 mm vs. 0.2 mm, *p* = 0.02). Greater tension resulted in less vertebral body growth and greater differential growth. Future studies should quantify forces applied during VBT surgery, as well as the forces maintained in the post‐operative period with spinal motion.

**Conclusion:**

Intraoperative intervertebral tensioning significantly affects vertebral body growth over 2 years.

## Graphical abstract

Intraoperative intervertebral tensioning significantly affects vertebral body growth over 2 years. Greater tension resulted in less vertebral body growth and greater differential growth. Further studies would benefit from better methods to quantify forces applied during surgery.

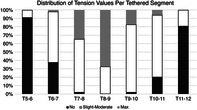

## 1 Introduction

Vertebral body tethering (VBT) has gained popularity as an alternative to posterior spinal fusion (PSF) for adolescent idiopathic scoliosis (AIS) [1, 2, 3, 4]. In contrast to PSF, VBT aims to not only improve deformity but also preserve the growth and flexibility of the spine [1, 2, 3, 4, 5]. To achieve this, a flexible cord is placed on the convex side of the curve and tensioned to achieve immediate correction [2, 3]. In the postoperative period, mechanical loading through the tensioned tether is thought to influence the rate of longitudinal growth of the vertebrae via the Hueter–Volkmann Law, which states that placing compressive loads on a physics results in a decreased growth rate [3, 6, 7].

VBT has been demonstrated in both animal models [8, 9, 10] and clinical studies [11, 12] to produce curve creation (animal models) or correction (clinical studies) via immediate alignment change and gradually induced growth modulation [2, 12, 13, 14, 15]. Newton et al. demonstrated with in vivo bovine and porcine models that spine tether placement induced growth modulation, generating significant coronal deformity [8, 16] and changes in vertebral body wedging [10].

However, the relationship between intraoperative tension and growth modulation has not been studied in growing children with spinal deformity. Additionally, the exact forces involved in spinal growth modulation have yet to be defined, and the appropriate amount of tension needed to be placed through the cord to induce growth modulation is unknown [3]. Currently, surgeons must estimate the necessary tension required to achieve the correction based upon the curve magnitude, curve flexibility, and estimated remaining growth of the child [3, 5, 7]. While several skeletal maturity scoring systems describe the estimated growth remaining and predict peak height velocity to aid in the correct timing of surgery, applying tension intraoperatively remains largely subjective and at the discretion of the treating surgeon.

The purpose of this study was to compare growth between the tethered and untethered side of individual vertebral bodies following VBT surgery with varying amounts of tether tension. We hypothesized that increased tension would correlate with decreased growth on the tethered side of the vertebral body, leading to greater differential height change over time.

## 2 Methods

IRB approval was obtained for this study (level of evidence: 3). A retrospective, multicenter pediatric spine deformity registry was queried for patients with a diagnosis of idiopathic scoliosis who were treated with right‐sided thoracic VBT between 2014 and 2018. For inclusion, patients were required to have a 2‐year follow‐up after surgery, intraoperative tether tension data recorded, and biplanar radiographs that allowed 3D spine reconstructions. All patients had major thoracic curve types. Intraoperative tension values for each tensioned level were recorded by the surgeon at the time of surgery. Skeletal maturity was determined retrospectively by rating triradiate cartilage status and proximal femoral maturity index (PFMI), as Sander's Maturity score was not routinely collected by the surgeons at the time the surgeries were performed [17]. As the PFMI was determined retrospectively, we chose to include all patients who met the inclusion criteria to fully assess how tension affects growth modulation across a wide range of growth potential. 3D measurements were taken from reconstructions created from simultaneous, biplanar radiographs at the 1st postoperative (first erect, FE) and 2‐year postoperative time points.

### 2.1 Surgical Technique

**FIGURE 1:**
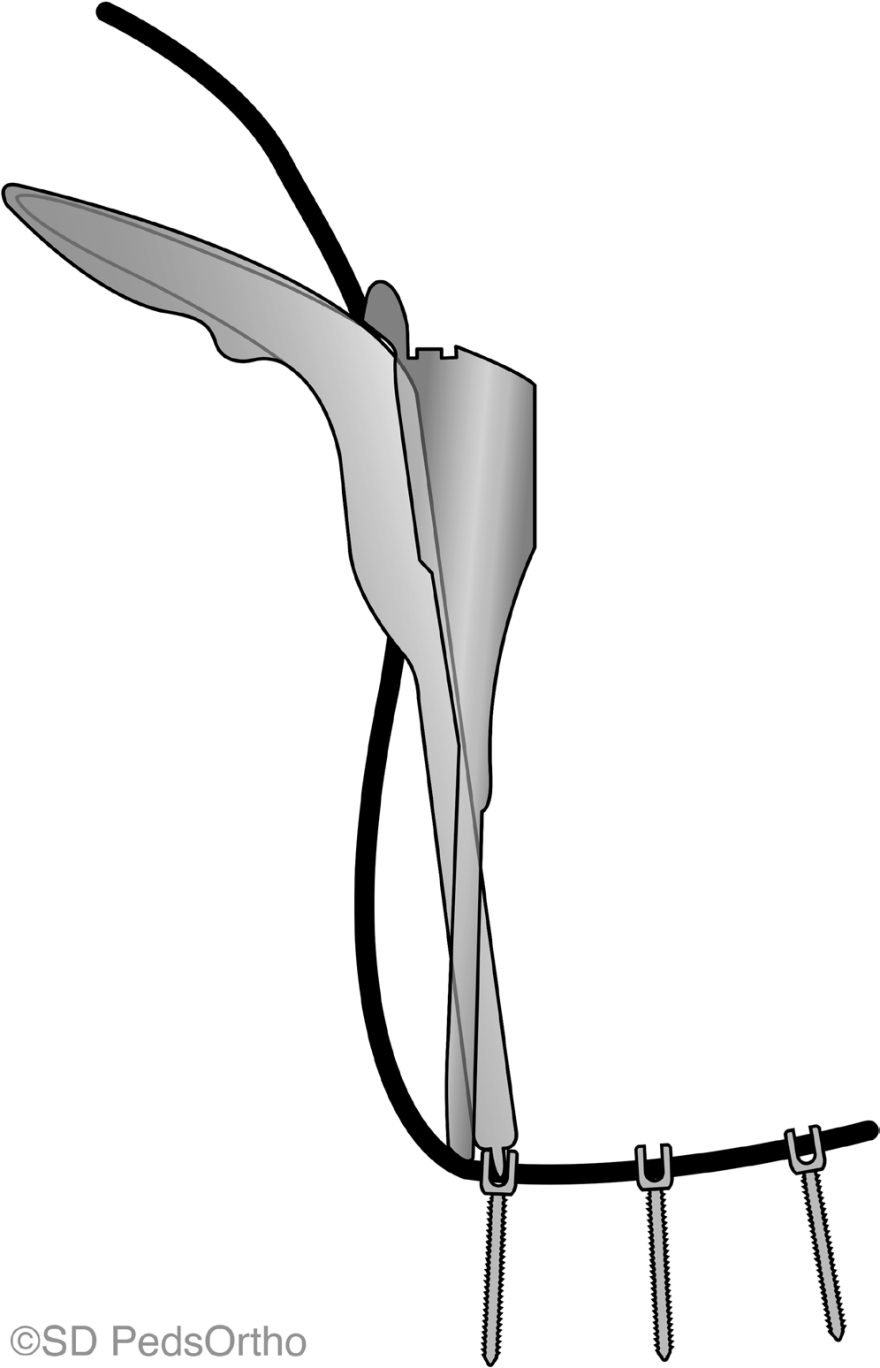
Intraoperative tether tension device, ZimVie Dynesis tether system (Warsaw, IN, USA).

**FIGURE 2:**
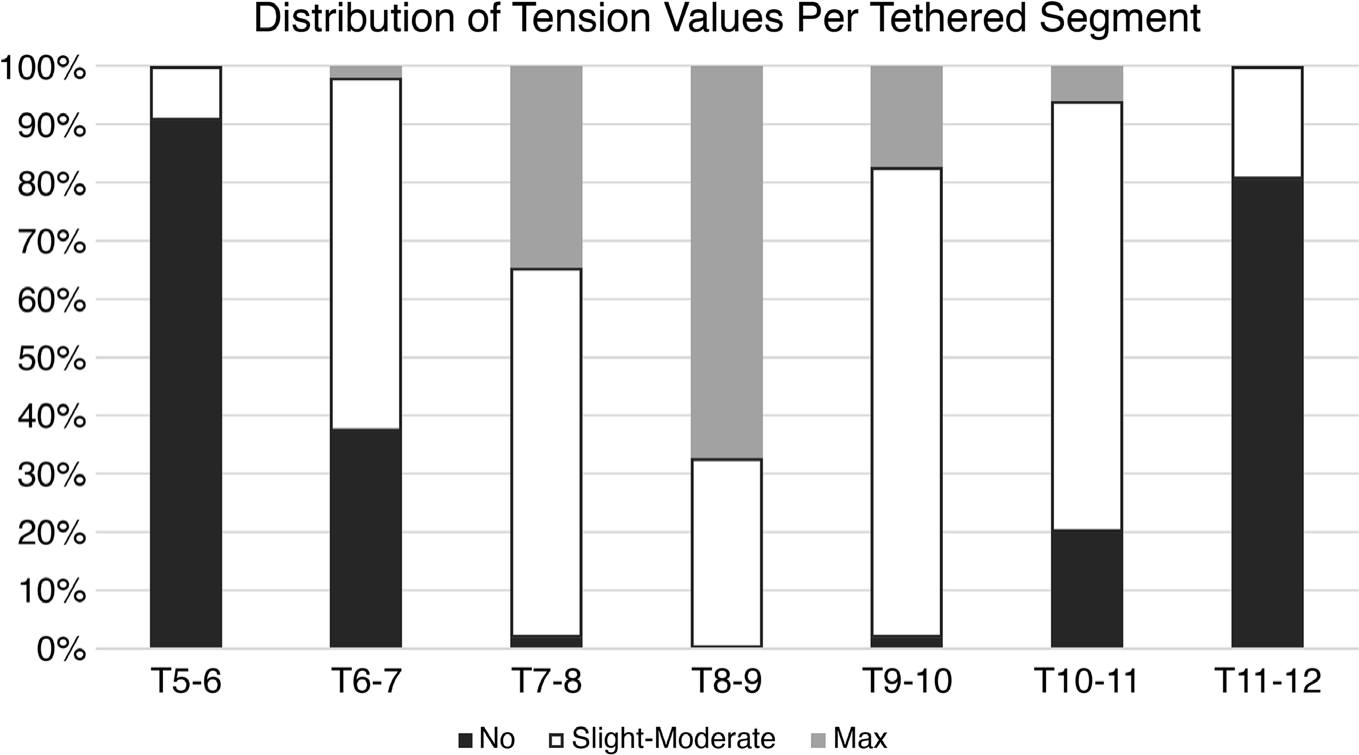
Proportion of tether segments with varying intraoperative tension values.

All patients underwent thoracoscopic right‐sided vertebral body tether procedures using the legacy Zimmer Biomet Dynesis tether system (Warsaw, IN, USA). The amount of intraoperative tension applied was at the discretion of the treating surgeon and depended on the patient's age, estimated growth remaining, desired amount of immediate correction, and/or specific curve pattern. All surgeries were performed at two sites by two senior surgeons. Tension was applied using the tensioning device included in the tether instrumentation set (Figure 1) and recorded by the surgeon at the time of surgery for each level on an ordinal scale from 0 to 3 (0 = no tension, 1 and 2 = slight‐moderate tension, 3 = maximum tension). Prior research has shown the reproducibility of quantifying the tension applied by various surgeons using this ordinal scale [18]. All tension measurements were recorded prospectively at the time of surgery. Most commonly, maximum tension is applied to the apex, with less tension at the periapical levels, and minimal to no tension applied at the upper and lower instrumented levels (Figure 2).

### 2.2 Radiographic Analysis

**FIGURE 3:**
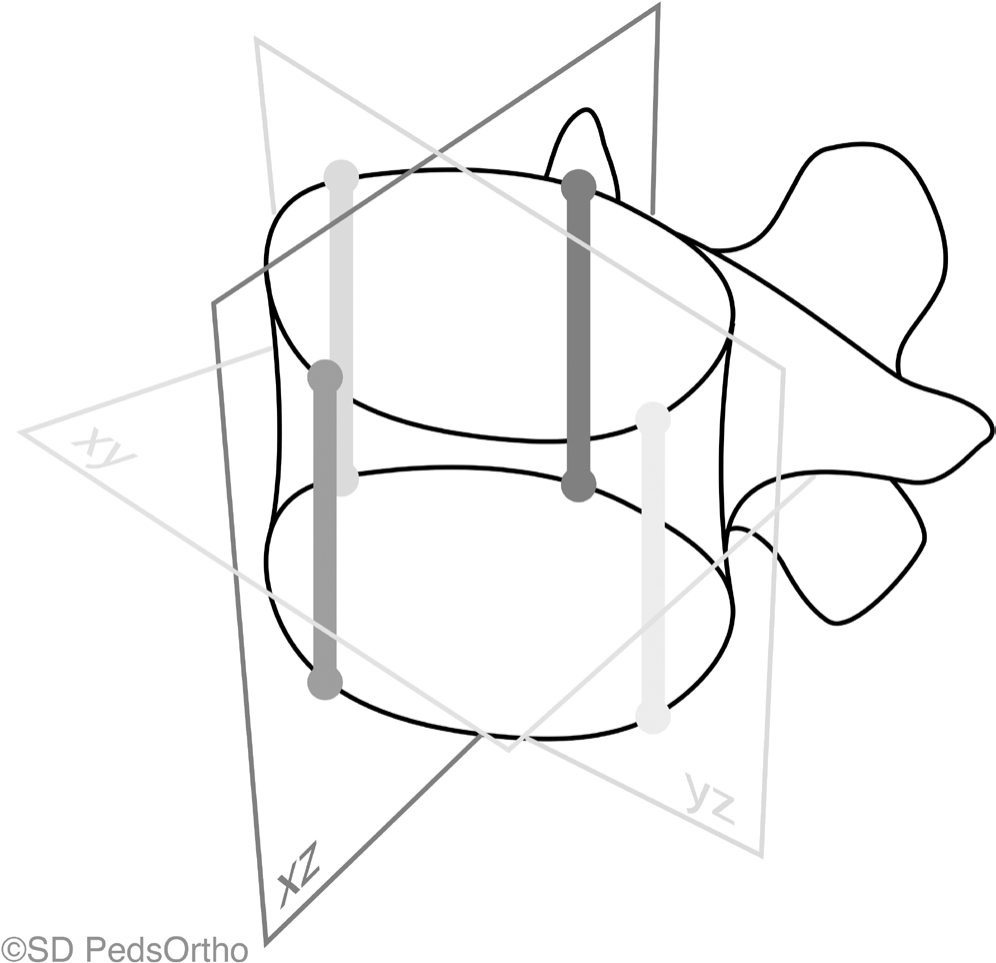
Graphic representation of vertebral height measurements. The lighter bars show the right, or convex side, height and the left, or concave side, height. The darker bars show the anterior height and the posterior height.

Postoperative radiographs were obtained at the FE and 2‐year postoperative time points. All patients had simultaneous, biplanar, slot scanning posteroanterior and lateral full‐length scoliosis radiographs obtained with an EOS imaging system, with 3D reconstructions created using sterEOS software (Alphatec Spine, Carlsbad, CA, USA). Custom MATLAB (MathWorks, Natick, MA, USA) software script was used to quantify the 3D vertebral body morphology from the spine reconstructions [19]. Prior studies have demonstrated the accuracy of 3D vertebral reconstructions using sterEOS to be within 1.1–1.5 mm [20, 21]. Vertebral height (mm) on the concave (untethered) and convex (tethered) sides of each vertebra, from T5 to T12, was calculated (Figure 3). These levels were chosen as they were the most consistently instrumented levels in this cohort. Radiographic outcomes (i.e., changes in curve magnitudes, proportion of patients achieving acceptable curve magnitude at 2 years) were not analyzed and were out of the scope of this study.

### 2.3 Statistics

Standard descriptive summaries (e.g., means and standard deviations for continuous variables) were used to summarize demographic variables. Comparisons of categorical variables between subgroups were made using the chi‐square test or Fisher's exact test, depending on the size of the sample. ANOVA was utilized for categorical variables for greater than two groups. Generalized linear mixed models (GLMM) with subject as a random factor were used to analyze the effect of tension on vertebral body growth over the postoperative period (utilizing the calculated change between FE and 2‐year measurements). Tension groups were utilized as a fixed factor in the model, and vertebral level above the tensioned segment was included in the model to account for the effect of level. Post hoc tests were performed between tension levels when the overall model detected significance. Alpha was set at *p* < 0.05 to declare significance. All statistics were performed with SPSS v28 (IBM Corp. Released 2021. IBM SPSS Statistics for Windows, Version 28.0. Armonk, NY: IBM Corp).

## 3 Results

Overall, 52 subjects were included. There were 47 females and 5 males. The mean age at the time of surgery was 12.5 ± 1 years. Most patients were skeletally immature at surgery. Triradiate cartilage was open in 23 (44%) patients. Thirty‐eight (73%) patients were Risser 0, 6 (12%) were Risser 1, and 8 (15%) were Risser 2–4. PFMI was 2 in 9 (17%) patients, 3 in 21 (40%) patients, 4 in 20 (38%) patients, and 5 in 2 (4%) patients. There was no significant difference in the distribution of skeletal maturity scores and the amount of tension applied per vertebral level (Table 1).

**TABLE 1:** Skeletal maturity score and distribution by tension category.

PFMI	Patients, *n*	No tension (*N* = 93 vertebra)	Slight‐moderate tension (*N* = 171 vertebra)	Max tension (*N* = 66 vertebra)	*p*
1	0	0 (0%)	0 (0%)	0 (0%)	0 0.896
2	9	15 (16%)	32 (19%)	10 (15%)	
3	21	36 (39%)	72 (42%)	25 (38%)	
4	20	39 (42%)	60 (35%)	29 (44%)	
5	2	3 (3%)	7 (4%)	2 (3%)	

Abbreviation: PFMI, proximal femoral maturity index.

A total of 330 vertebral bodies were analyzed (Table 2). No significant difference was found in average concave (untethered side) height change based on tension (2.0 vs. 1.7 vs. 1.5 mm, *p* = 0.2) for no, slight‐moderate, and max tension at 2 years, respectively. On the convex side, greater tension resulted in less height change (1.7 vs. 1.1 vs. 0.5 mm, *p* = 0.002) for no, slight‐moderate, and max tension, respectively. The GLMM accounts for the effect of the patient (including age and skeletal maturity) as well as the effect of the individual level.

**TABLE 2:** Summary of vertebral body height change by tension category.

	No tension (*N* = 93 vertebrae)	Slight‐moderate tension (*N* = 171 vertebrae)	Max tension (*N* = 66 vertebrae)	*p*	Post hoc *p*
Concave height change (mm)	2.0 ± 0.2	1.7 ± 0.2	1.5 ± 0.2	0.2	N/A
Convex height change (mm)	1.7 ± 0.2	1.1 ± 0.2	0.5 ± 0.2	0.002*	No tension vs. slight‐moderate: 0.03* No tension vs. maximal: < 0.001* Slight‐moderate vs. maximal: 0.04*
Differential height change (concave–convex) (mm)	0.2 ± 0.2	0.6 ± 0.2	0.8 ± 0.2	0.044*	No tension vs. slight‐moderate: 0.1 No tension vs. maximal: 0.02* Slight‐moderate vs. maximal: 0.5

* Statistically significant value (*p* < 0.05).

In post hoc analysis, the difference between all tension groups was statistically significant (*p* ≤ 0.04) for the mean concave vertebral growth. Additionally, greater tension resulted in greater differential growth between the convex and concave sides of the vertebra (0.2 vs. 0.6 vs. 0.8 mm, *p* = 0.044) for no, slight‐moderate, and max tension, respectively. The post hoc test revealed that a significant difference was observed between maximal tension compared with no tension (0.8 vs. 0.2 mm, *p* = 0.02). The difference between no tension and slight‐moderate did not reach significance (*p* = 0.10), nor did the difference between the slight‐moderate vs. max tension comparison (*p* = 0.5).

## 4 Discussion

VBT offers a non‐fusion alternative to PSF for growing patients with progressive AIS [2, 3]. However, questions remain regarding the amount of proper tether tensioning required to produce reliable growth modulation. Animal studies have demonstrated the ability of distraction and compression applied to growing bones to affect the rate of growth [10, 15]. Aronsson found that vertebrae of bovine tails loaded in compression had a growth rate of 68% compared to controls, whereas the vertebrae loaded in distraction had a growth rate of 123% [15]. However, there are limited data on the role of differential tensioning on growth modulation in clinical studies of children treated with VBT. A prior study, using 2D radiographic measurements, demonstrated segmental vertebral body changes following VBT [12]. McDonald et al. showed vertebral body height change on the tethered side of the spine relative to the untethered side (2.0 vs. 1.5 mm) [12]. The findings from our cohort of largely skeletally immature patients treated with VBT further support this, with the additional finding that the amount of intervertebral tension applied intraoperatively had a statistically significant effect on vertebral body growth differential over 2 years following the VBT procedure. Photopoulos et al. [22] demonstrated maintenance of differential growth in periapical vertebral bodies at 4 years following VBT. However, this study relied on standard radiographs and was unable to account for segmental tether tension [22]. Additionally, we found that greater tension resulted in less convex‐sided vertebral growth, leading to greater differential growth between the concave and convex sides of the vertebra. Our study relied on 3D measurements obtained from biplanar radiographs. However, both McDonald [12] and Photopoulos [22] relied on 2D images using a combination of slot‐scanning biplanar radiographs and standard radiographs. This difference in methodology may have led to the different findings, as a true lateral projection of the spine is difficult to obtain without 3D analysis given the rotational aspect of the spine deformity. The Photopoulos paper was written in collaboration with the Pediatric Spine Study Group, while the McDonald paper was in collaboration with the Harms Study Group. However, there was substantial overlap, as many patients were enrolled in both study groups.

The ability to generate enough tension to induce growth modulation of the spine is an important component of the surgery [2, 3]. However, quantifying the tension has been difficult and largely relies on surgeon experience to determine the correct amount of tension and must take into consideration the age of the patient, the amount of growth remaining, the curve magnitude, the curve flexibility, and the desired immediate curve correction [3]. A larger, stiffer curve in an older patient may require more tension compared to a small, flexible curve in a younger patient. However, currently there is no known defined adequate tension, and the inability to quantify the tension intraoperatively makes this difficult to study and assess. The vertebral body tether system used in this study appears capable of generating adequate tension given the observed ability to produce growth modulation, but precise quantification of that tension remains a challenge. This is especially true once the patient is erect and the mechanical loads on the spine and the intervertebral discs are influenced by motion and gravity. However, prior benchtop mechanical testing has demonstrated that surgeons were able to reproduce the level of tension using standard VBT instrumentation with high inter‐ and intra‐rater reliability [18]. The development of intraoperative tensiometers will be necessary to provide objective measures to further study the appropriate amount of tension to use at the time of tether application.

The tension must be calibrated to induce growth modulation while avoiding complications. Too much compression may damage the cartilage and the interposed intervertebral disc [23] or increase the risk of tether rupture or overcorrection [2, 24]. While there remains concern for possible accelerated disc degeneration in response to increased spine compression [23] previous animal [8, 25] and clinical studies [26] have not shown adverse effects on disc health following VBT. On the other hand, inadequate compression may allow continued growth with progression of the spinal deformity [24, 27]. While the patient must have enough growth remaining to allow for growth modulation [28], tethering at a young age has been associated with complications such as tether rupture and overcorrection [24, 27, 28, 29]. Further questions remain regarding the durability of outcomes in the absence of growth modulation. Clinical success is often defined as the child reaching skeletal maturity with a major curve magnitude of < 30°–35° regardless of growth modulation or the presence or absence of a known or suspected ruptured tether [13, 27]. Better understanding of the forces generated by the tether construct should improve our ability to provide more consistent and reliable outcomes.

Limitations of this study include limited generalizability given a relatively small sample size. Relying on qualitative tether tension values limits the reproducibility of the findings or the ability to make specific recommendations about tension values per level. Tether tension values were unable to be quantified and relied on the surgeon's subjective evaluation of the amount of tension applied at each level. Additionally, this may not account for additional adjunctive maneuvers used to tension the cord [3, 5]. More objective measures would be useful to establish tension threshold values necessary to induce growth modulation. However, these data do support the hypothesis that increased tension leads to a greater effect on growth modulation. Additionally, this study does not evaluate changes at the level of the intervertebral disc, such as disc wedging or disc height changes with time, which may have complex interactions with the overall change in curve magnitude. Prior studies have shown counterintuitive alterations in disc wedging in response to tether tension [16]. Newton et al. found in a porcine model that the disc wedging following tethering opened up towards the tethered side [16]. Our study focused only on the vertebral body growth of the instrumented levels. Clinical outcomes may be largely affected by factors such as the behavior of the instrumented curve or other patient factors unrelated to growth modulation. As the scope of this study was to primarily evaluate how tension affects the growth modulation of the instrumented vertebrae, we chose not to evaluate clinical outcomes specifically. Further studies are necessary to evaluate the overall changes in curve magnitude or changes in the uninstrumented spine. Other patient‐specific factors that may affect vertebral body growth were also not accounted for, such as Sanders skeletal maturity score [30], which was not routinely utilized at the time of the surgeries. Rather, the triradiate cartilage status and PFMI were retrospectively reviewed to assess skeletal maturity.

The current study builds on the prior understanding of the effects of VBT on growth modulation of the spine by demonstrating the effect of various levels of tether tension on the rates of growth in human subjects via a 3D analysis of the growing spine. We found that intraoperative intervertebral tensioning had a significant impact on vertebral body growth over 2 years. Greater tension resulted in less convex‐side vertebral body growth and greater differential growth between the concave and convex sides of the vertebra. Further studies would benefit from better methods to quantify forces applied during surgery.

## Author Contributions

**Taylor J. Jackson:** conceptualization, formal analysis, writing – original draft, writing – review and editing. **Craig Louer:** conceptualization, formal analysis, writing – original draft, writing – review and editing. **Ron El‐Hawary:** conceptualization, formal analysis, writing – original draft, writing – review and editing. **Jennifer Hurry:** conceptualization, formal analysis, writing – original draft, writing – review and editing. **Hui Nian:** conceptualization, formal analysis, writing – original draft, writing – review and editing. **Christine Farnsworth:** conceptualization, formal analysis, writing – original draft, writing – review and editing. **Carrie E. Bartley:** conceptualization, formal analysis, writing – original draft, writing – review and editing. **Tracey P. Bryan:** conceptualization, formal analysis, writing – original draft, writing – review and editing. **Michael P. Kelly:** conceptualization, formal analysis, writing – original draft, writing – review and editing. **Peter O. Newton:** conceptualization, formal analysis, writing – original draft, writing – review and editing. **Stefan Parent:** conceptualization, formal analysis, writing – original draft, writing – review and editing. **Pediatric Spine Study Group:** conceptualization, formal analysis, writing – original draft, writing – review and editing. **Vidyadhar V. Upasani:** conceptualization, formal analysis, writing – original draft, writing – review and editing. All authors have read and approved the final submitted manuscript.

## Conflicts of Interest

Taylor J. Jackson, MD: Funding for this work was supported in part by the Scoliosis Research Society Biedermann Innovation Award and the 2022 POSNA Zimmer Biomet Award. Craig Louer, MD: Funding for this work was supported in part by the Scoliosis Research Society Biedermann Innovation Award and the 2022 POSNA Zimmer Biomet Award. Disclosures outside of the submitted work include: Depuy Synthes Spine: Consultant, Globus/Nuvasive Spine: Research support; Educational Grant (paid to institution), National Scoliosis Clinics Inc.: Royalties, POSNA: Board of Directors; Research Support; Zimmer Biomet Spine Research Grant. Ron El‐Hawary, MD: Funding for this work was supported in part by the Scoliosis Research Society Biedermann Innovation Award and the 2022 POSNA Zimmer Biomet Award. Disclosures outside of the submitted work include: Children's Spine Foundation: Board or committee member, DePuy, A Johnson & Johnson Company: Paid consultant; Institutional research support, Medtronic: Paid consultant; research support, Orthopediatrics: Paid consultant; Paid presenter or speaker; Stock or stock options, Pediatric Spine Study Group: Board or committee member, Scoliosis Research Society: Board or committee member, Springer: Publishing royalties, financial or material support, Zimmer: Research support. Jennifer Hurry, MASc, PEng: Funding for this work was supported in part by the Scoliosis Research Society Biedermann Innovation Award and the 2022 POSNA Zimmer Biomet Award. Disclosures outside of the submitted work include: DePuy, A Johnson & Johnson Company: Institutional research support. Hui Nian, PhD: Funding for this work was supported in part by the Scoliosis Research Society Biedermann Innovation Award and the 2022 POSNA Zimmer Biomet Award. Christine Farnsworth, MS: Funding for this work was supported in part by the Scoliosis Research Society Biedermann Innovation Award and the 2022 POSNA Zimmer Biomet Award. Carrie E. Bartley, MA: Funding for this work was supported in part by the Scoliosis Research Society Biedermann Innovation Award and the 2022 POSNA Zimmer Biomet Award. Tracey P. Bryan, MA: Funding for this work was supported in part by the Scoliosis Research Society Biedermann Innovation Award and the 2022 POSNA Zimmer Biomet Award. Michael P. Kelly, MD, MSc: Funding for this work was supported in part by the Scoliosis Research Society Biedermann Innovation Award and the 2022 POSNA Zimmer Biomet Award. Disclosures outside of the submitted work include: AO Spine: Payment to support attendance at meetings and/or travel; board or committee member, Cervical Spine Research Society: Board or committee member, San Diego Spine Foundation: Grants, Scoliosis Research Society: Grants; board of directors, Spine: Editorial or governing board. Peter O. Newton, MD: Funding for this work was supported in part by the Scoliosis Research Society Biedermann Innovation Award and the 2022 POSNA Zimmer Biomet Award. Disclosures outside of the submitted work include: DePuy Synthes Spine: Grant paid to institution/research support, royalties or licenses paid to me, consulting fees, speakers bureau, payment for development of educational programs, patents: Anchoring systems and methods for correcting spinal deformities (8540754); Low profile spinal tethering systems (8123749); screw placement guide (79811117); compressor for use in minimally invasive surgery (7189244), Scoliosis Research Society: Grant paid to institution, EOS Imaging: Grant paid to institution, Medtronic: Payment or honoraria for lectures, presentations, speaker bureaus, manuscript writing, or educational events, paid to me, Nuvasive: Grant paid to institution, Orthopediatrics: Grant paid to institution, Stryker/K2M: Grant paid to institution, royalties or licenses paid to me, consulting fees, patent: Posterior spinal fixation, Alphatech: Grant paid to institution, Setting Scoliosis Straight Foundation: Grants paid to institution, board membership (unpaid), Thieme Publishing: Royalties or licenses paid to me, Globus Medical: Consulting fees, Pacira: Consulting fees, International Pediatric Orthopedic Think Tank: Board membership (unpaid), Rady Children's Specialists of San Diego: Board membership (unpaid), Accelus: stock or stock options, Spinology: Stock or stock options. Stefan Parent, MD, PhD: Funding for this work was supported in part by the Scoliosis Research Society Biedermann Innovation Award and the 2022 POSNA Zimmer Biomet Award. Disclosures outside of the submitted work include: Canadian Institute of Health Research: Grants, Canadian Spine Society: Board or committee member, DePuy, A Johnson & Johnson Company: Grants; Paid consultant; Paid presenter or speaker; Research support (Academic Research Chair in spine deformities of the CHU Sainte‐Justine [endowments]); fellowship support, EOS‐Imaging: Grants; IP royalties; Consulting fees; Research support, Fonds de recherche Québec—Santé: Grants, K2M: Consulting fees, Natural Sciences and Engineering Council of Canada: Grants, Medtronic: Fellowship support, Orthopediatrics: Paid presenter or speaker; Fellowship support, Pediatric Orthopaedic Society of North America: Grants, Scoliosis Research Society: Board or committee member; Grants, Setting Scoliosis Straight Foundation: Research support, Spinologics: Co‐founder of company; Employee; Stock or stock Options. Pediatric Spine Study Group: Funding for this work was supported in part by the Scoliosis Research Society Biedermann Innovation Award and the 2022 POSNA Zimmer Biomet Award. Disclosures outside of the submitted work include: Pediatric Spine Study Group is supported by the Pediatric Spine Foundation (PSF). PSF receives research funding from: DePuy Synthes Spine, Globus, OrthoPediatrics, ZimVie, Educational support: ZimVie, DePuy Synthes, OrthoPediatrics, Medtronic, Globus, nView Medical, Stryker, Atec, Boston Orthotics, Pacira. Vidyadhar V. Upasani, MD: Funding for this work was supported in part by the Scoliosis Research Society Biedermann Innovation Award and the 2022 POSNA Zimmer Biomet Award. Disclosures outside of the submitted work include: AAOS: Board or committee member, Daedalus Medical Solutions Inc.: Patents (planned, issued, or pending); Employee, DePuy, A Johnson & Johnson Company: Paid consultant, Imagen: Stock or stock Options, Indius: unpaid consultant, nView: Research support, Orthofix Inc.: Royalties or licenses; paid consultant, OrthoPediatrics: IP royalties; paid consultant; research support, Pacira: Paid consultant, Pediatric Orthopaedic Society of North America: Board or committee member (unpaid), Scoliosis Research Society: Board or committee member (unpaid), Spine Journal: Editorial or governing board (unpaid), Stryker: Paid consultant, Wolters Kluwer Health–Lippincott Williams & Wilkins: Publishing royalties, financial or material support, ZimVie: Research support.
